# Effect of subsequent vertebral body fractures on the outcome after posterior stabilization of unstable geriatric fractures of the thoracolumbar spine

**DOI:** 10.1186/s12891-022-06031-z

**Published:** 2022-12-05

**Authors:** U. J. Spiegl, J.-S. Jarvers, G. Osterhoff, P. Kobbe, P.-L. Hölbing, K. J. Schnake, C.-E. Heyde

**Affiliations:** 1 Department of Orthopaedics, Trauma Surgery and Plastic Surgery, University Hospital Leipzig, Leipzig, Germany; 2 Department of Trauma and Reconstructive Surgery, University of Aachen, Aachen, Germany; 3 Center for Spinal and Scoliosis Surgery, Malteser Waldkrankenhaus St. Marien, Erlangen, Germany; 4 Department of Orthopedics and Traumatology, Paracelsus Private Medical University Nuremberg, Nuremberg, Germany

**Keywords:** Osteoporotic vertebral body fractures, Trauma mechanism, Thoracolumbar spine, Reduction loss, Subsequent fractures, Posterior stabilization

## Abstract

**Purpose:**

The purpose of this study was analyzing the effect of subsequent vertebral body fractures on the clinical outcome in geriatric patients with thoracolumbar fractures treated operatively.

**Methods:**

Retrospectively, all patients aged ≥ 60 with a fracture of the thoracolumbar spine included. Further inclusion parameters were acute and unstable fractures that were treated by posterior stabilization with a low to moderate loss of reduction of less than 10°. The minimal follow-up period was 18 months. Demographic data including the trauma mechanism, ASA score, and the treatment strategy were recorded. The following outcome parameters were analyzed: the ODI score, pain level, satisfaction level, SF 36 score as well as the radiologic outcome parameters.

**Results:**

Altogether, 73 patients were included (mean age: 72 years; 45 women). The majority of fractures consisted of incomplete or complete burst fractures (OF 3 + 4). The mean follow-up period was 46.6 months. Fourteen patients suffered from subsequent vertebral body fractures (19.2%). No trauma was recordable in 5 out of 6 patients; 42.8% of patients experienced a low-energy trauma (significant association: *p* < 0.01). There was a significant correlation between subsequent vertebral body fracture and female gender (*p* = 0.01) as well as the amount of loss of reduction (*p* = 0.02). Thereby, patients with subsequent vertebral fractures had significant worse clinical outcomes (ODI: 49.8 vs 16.6, *p* < 0.01; VAS pain: 5.0 vs 2.6, *p* < 0.01).

**Conclusion:**

Patient with subsequent vertebral body fractures had significantly inferior clinical midterm outcome. The trauma mechanism correlated significantly with both the rate of subsequent vertebral body fractures and the outcome. Another risk factor is female gender.

## Introduction

The optimal therapy of osteoporotic vertebral body fractures of the thoracolumbar spine has been discussed controversially [1]. Reported clinical outcomes vary widely both after conservative and operative treatment [1–6]. In the majority of patients non-operative treatment is recommended [1]. However, several risk factors for non-operative treatment have been reported [7]. Relevant kyphotic malalignment leads to an increase rate of subsequent vertebral body fractures due to increased biomechanical stress first of all at the adjacent vertebral bodies and secondly in the midthoracic spine [8]. However, studies have shown unexpected high rates of subsequent vertebral body fractures after posterior stabilization despite low to moderate loss of reduction [6, 9]. Additionally, subsequent vertebral fractures were associated with worse clinical outcome after operative stabilization [6]. However, this was not evaluated systematically.

Therefore, the first aim of this study was to evaluate the effect of subsequent vertebral body fractures on the clinical midterm outcome in geriatric patients who suffered from a single unstable thoracolumbar fracture stabilized by posterior instrumentation with low to moderate reduction loss of less than 10°. The second aim was to analyze risk factors for the development of subsequent vertebral body fractures. We hypothesized that subsequent vertebral fractures would have a negative effect on the clinical outcome and that the trauma mechanism may have an impact on the rate of subsequent vertebral body fractures.

## Methods

The study was performed retrospectively at a single level I spine center between January 2010 and December 2017. All geriatric patients (aged 60 and older) with acute unstable vertebral body fractures of the thoracolumbar spine were evaluated. Further inclusion parameters were operative treatment with posterior stabilization, anatomic reduction of the bisegmental kyphosis (defined as less than 5° difference to the average of the one level above and one level below), a minimal follow-up period of 18 months, and low to moderate rate of loss of reduction of less than 10°. Further inclusion criteria were a single vertebral body fracture. All fractures were classified in accordance with the OF-classification [10]. Unstable fracture morphology was defined in patients with vertebral fractures of OF type of 4 and 5 as well as OF type 3 fractures with a bisegmental loss of reduction of more than 5° after mobilization. Generally, the indication for surgery was seen in accordance to Blattert et al. [11] in patients with an OF score of 6 and higher. Patients with any neurologic deficits or pathologic fractures due to malignancies were excluded. The study was approved by the institutional ethics committee. Thereby, the study was performed in accordance with relevant guidelines and regulations. An informed consent was contained from all patients.

Initially, whole body computer tomography (CT) was performed in patients suffering from high energy accidents, all others received conventional radiographs. Magnetic resonance imaging (MRI) of the whole spine was performed in those patients without MRI contraindications. Otherwise, an additional CT was carried out in patients with signs of vertebral fractures after low and moderate energy trauma, which was done in 4% of the patients. The trauma mechanism was analyzed and divided into low energy trauma, moderate energy trauma, and high energy trauma in accordance to Spiegl et al. [9]. Additionally, fractures that could not be remembered were defined as insufficiency fractures. Low-energy trauma was defined as stumbling while walking or falling while standing. Moderate energy trauma was defined as traffic accidents with low velocity (≤ 30 km/h) and falls above standing height and less than 3 m, whereas high energy trauma was defined as falls from height of greater than 3 m and car accidents with higher velocities (> 30 km/h).

The (ASA) scale was obtained based in all patients [12].

### Surgical techniques

Posterior stabilization was done via a minimally invasive or open midline approach in accordance to Spiegl et al. [6, 9]. Posterior stabilization was done with cement-augmented pedicle screws (Matrix, Fa. DePuy-Synthes; Viper, Fa. DePuy-Synthes; USS 2; Fa. DePuy-Synthes; Longitude, Fa. Medtronic, MUST, Fa. Medacta). The approach and the implant were chosen as preferred by the surgeon. All patients with fractures of the thoracolumbar junction and the lumbar spine (Th11 – L4) were treated with short-segmental posterior stabilization either with kyphoplasty of the fractured vertebral body or with a vertebral body replacement by an additional anterior approach. The majority of patients with midthoracic fractures were treated by posterior long-segmental stabilization including two vertebral bodies above and below the fracture with or without kyphoplasty of the fractured vertebral body.

### Postoperative management

Postoperative management was done in accordance to Spiegl et al. [6, 9]. All patients received conventional standing radiographs. An additional CT scan was taken in cases of uncertainty of correct screw placement or anatomic reduction or in symptomatic patients. No brace or corset was used postoperatively. Physiotherapy was initiated on the day after surgery to improve mobility and muscle strength. Clinical and conventional standing radiological assessment was performed at 2-weeks, 6-weeks, 3-months, and 12-months postoperatively. Dual X-Ray Absorptiometry (DXA) assessment and sufficient anti-osteoporotic therapy were recommended to all patients.

### Outcome parameters

Patients were invited for clinical and radiological evaluation. An anterior–posterior radiograph centered on the injured vertebral body and lateral 36 inch views while standing were performed. The primary clinical parameter of interest was the Oswestry Disability Index (ODI) at last follow-up. Secondary outcome parameters were the level of pain (VAS 0–10 scale; 0: no pain, 10: maximal pain), level of satisfaction (VAS 0–10 scale; 0: lowest satisfaction, 10: highest), the SF-36 score (physical summary component and mental summary component), the complication rates, the rate of subsequent vertebral body fractures, and the rate of surgical revisions, that occurred during the postoperative course up to the latest follow-up. In addition, radiological parameters were measured, including the loss of reduction measured by the bisegmental Cobb angle, and the parameters pelvic tilt, pelvic incidence, sacral slope, and the lumbar lordosis. These were defined as tertiary outcome parameters.

### Statistics

Statistical analyses were performed using standardized SPSS software 25.0 (SPSS®, Inc. Chicago, USA). Statistical analysis was made using descriptive statistics. Two-sample Wilcoxon signed-rank tests were employed to compare the rate of subsequent fractures and all potential risk factors such as the trauma mechanism, fracture location, fracture classification, the treatment strategy, and the reduction loss between the postoperative and latest radiological parameters. After an exploratory data analysis, selected parameters were further investigated by Fisher`s exact test and Pearson`s test. Additionally, a multivariate analysis was performed (ANOVA). A significance level of 0.05 was used.

## Results

**Fig. 1:**
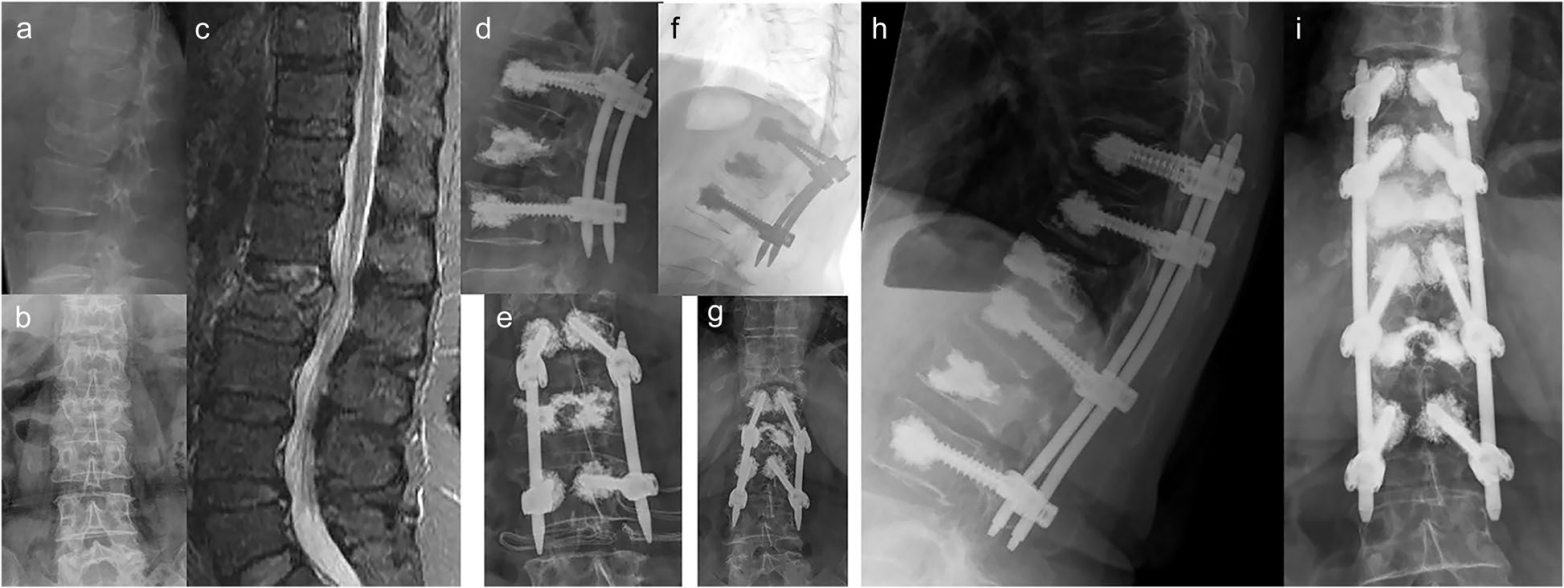
A case with a subsequent vertebral fracture is presented. A 72 year old patient suffered from an acute L2 fracture type OF 4 while helping to lift her husband out of the bed 8 days ago **a**-**c**. This trauma mechanism was defined as not adequate. Based on the intense pain despite adequate analgetig medication hybridstabilization consisting of percutaneous short-segmental posterior stabilization and cement augmentation of the fractured vertebral body was performed **d**,**e**. Acute pain occurred four months after having an eventful initial course. There was no trauma history. The radiographs depicted an subsequent fracture of the thoracic vertebral body 12 with kyphotic malalignment **f**,**g**. This was treated with long-segmental stabilization and cement-augmentation of the fractured vertebral body. The latest radiographs 6 years after the initial fracture are shown **h**,**i**

**Fig. 2:**
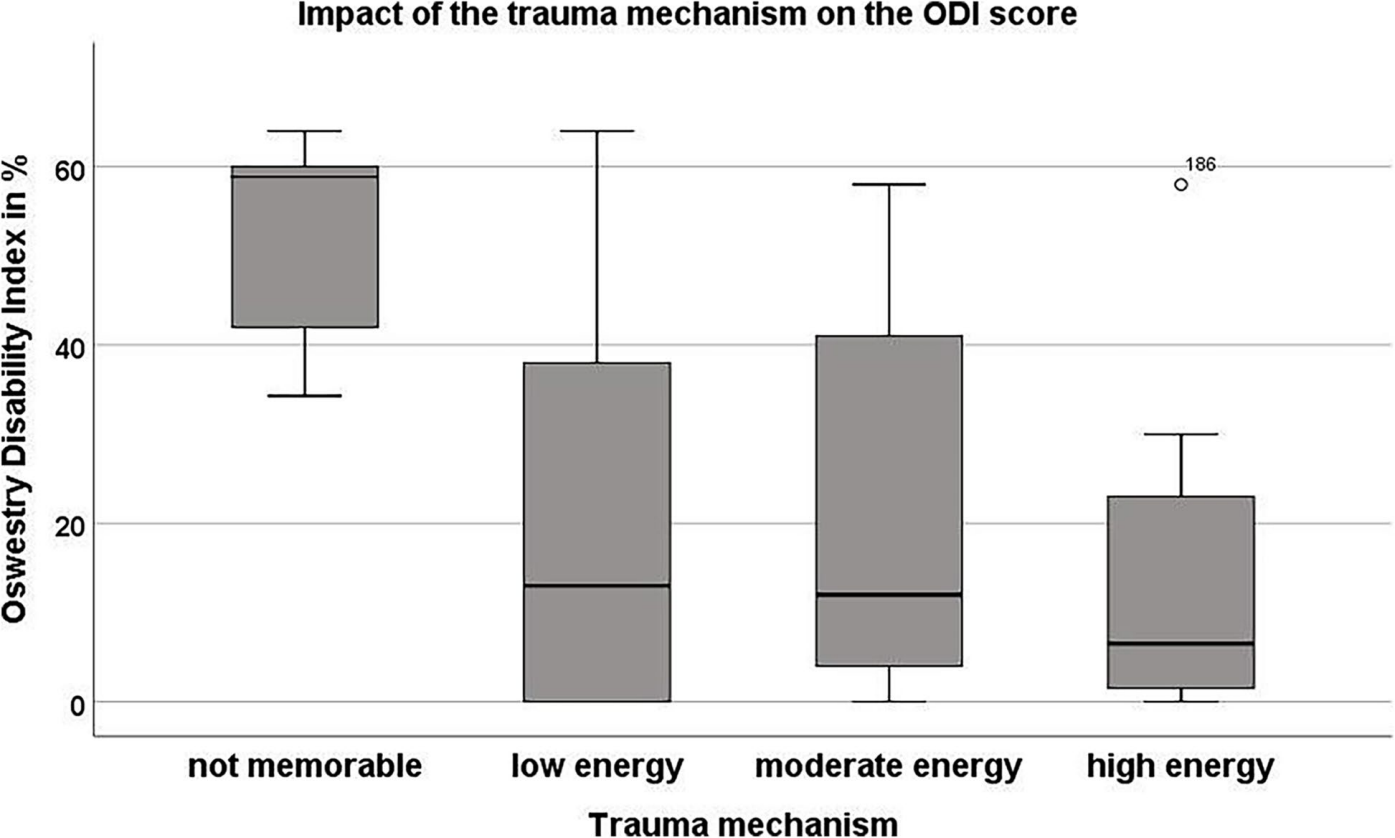
Box plot depicting the ODI scores of all included patients in dependence on the trauma mechanism

A total of 73 patients were included (Table 1). The average age was 72 years (range 60 to 86 years) including 45 (61.6%) women and 28 men (38.4%). The trauma mechanism could not be remembered in six patients (8.2%). Eleven patients (15.1%) had a low energy trauma, 39 (54.4%) experienced a moderate energy trauma, whereas 17 patients (23.3%) suffered from a high energy trauma. A total of 58.8% of the fractures occurred at thoracolumbar junction (Th 11 to L2). Thereby, fractures of L1 dominated (17.4%). Most fractures were incomplete burst fractures with a relevant injury of the posterior cortex (OF 3: *n* = 47; 64.4%), less frequently complete burst fractures of type OF 4 (*n* = 18: 24.7%) or OF 5 fractures (*n* = 8; 11.0%). The mean follow-up was 46.6 months (range: 18 – 111 months). Fourteen patients had a subsequent vertebral body fracture (19.2%). In seven patients (9.6%) the adjacent vertebral body with respect of the instrumentation and in the other 7 patients none-adjacent vertebral bodies (higher distance) were affected (Fig. 1). There was a significant correlation between the occurrence of subsequent fractures and the pain level (r = 0.32; *p* = 0.01), the ODI score (r = 0.41; *p* < 0.01), and the PSC of the SF 36 (r = -0.32; *p* = 0.01). Generally, patients with subsequent vertebral body fractures experience statistically significant higher ODI scores and suffered from more intense pain level (Table 2). Additionally, the occurrence of subsequent vertebral body fractures was significantly related to female gender (*p* = 0.01) and the reduction loss (*p* = 0.02) without any statistically significant differences in the follow-up time (*p* = 0.23). The mean loss of reduction was 6.1° in patients with subsequent fractures and 4.0° in patients without subsequent fractures. Five out of six patients with no remembered trauma suffered from a subsequent vertebral body fracture (83%). Similarly, the rate of subsequent fractures was higher in patients who suffered initially of low-energy trauma (42.8%) than those after moderate (14.3%) or high-energy (5.9%) trauma. There was a significant correlation between the trauma mechanism and the rate of subsequent vertebral body fractures (r = 0.49; *p* < 0.01). Considering the trauma history, only patients without remembered trauma were associated with significantly worse ODI scores (Fig. 2). There were no further statistically significant differences between patients with or without subsequent vertebral body fractures with respect to fracture location, fracture classification, treatment strategy, as well as surgical approach, and all alignment parameters (Table 2, Table 3). The multivariant analysis proofed that gender, trauma mechanism, and reduction were independent risk factors with significant impact on the generation of subsequent fractures (*p*-values: ˂0.001; ˂ 0.001; = 0.047, respectively).

**Table 1:** Patient collective

Parameter	Patient collective (*n* = 73)		
	Mean	Range	Std
Age	71.7	60—86	6.5
Follow-up period [months]	46.6	18 – 111	23.8
Female gender	61.6%		
Trauma mechanism (n)	not remembered: 6 low energy: 11 moderate energy: 39 high energy: 17		
Fracture location (n)	Th 3: 2 Th 4: 3 Th 5: 1 Th 6: 3 Th 7: 5 Th 8: 4 Th 9: 3 Th 10: 3 Th 11: 3 Th 12:10 L 1: 20 L 2: 10 L 3: 6		
Classification (n)	OF 3: 47 OF 4: 18 OF 5: 8		
Therapy strategy (n)	hybrid stabilization: 45 posterior only: 21 anterior–posterior: 7		
Stabilized segments	2 segments: 54 3–5 segments: 17 > 5 segments: 2		
Min. inv. approach	68.5%		

*Std* standard deviation; fracture location, *n* number, *Min. inv* minimal invasive

**Table 2:** Patients’ outcomes in dependence on the occurrence of subsequent vertebral body fractures

Parameter	Subsequent fractures (*n* = 14)		No subsequent fractures (*n* = 59)		*p*-value
	mean	Std	mean	Std	
ODI	49.8	13.4	16.6	17.5	** < 0.01**
PSC (SF-36)	24.5	5.9	39.1	12.0	** < 0.01**
Pain [NRS]	5.0	1.5	2.6	2.0	** < 0.01**
Treatment satisfaction [NRS]	6.8	1.7	7.8	2.3	0.15
Reduction loss [°]	6.1	2.9	4.0	2.9	**0.02**
Lumbar lordosis [°]	43.1	17.4	42.4	11.9	0.90
Sacral slope [°]	42.0	11.5	36.5	12.6	0.32
Pelvic tilt [°]	21.7	7.9	20.9	8.5	0.83

*STD* Standard deviation, *ODI* Oswestry disability index, *PSC* Physical summary score, *NRS* Numeric rating score, *R* Rate, *vert* vertebral

**Table 3:** Patients’ demographic data in dependence on the occurrence of subsequent vertebral body fractures

Parameter	Subsequent fractures (*n* = 14)		No subsequent fractures (*n* = 59)		*p*-value
	mean	Std	mean	Std	
Age	74.4	5.9	71.1	6.5	0.09
Female gender [%]	54.2		92.9		**0.01**
Trauma mechanism	1.1	1.0	2.1	0.7	** < 0.01**
Fracture location	6.3	3.5	5.9	4.8	0.78
Fracture classification	3.5	0.8	3.3	0.9	0.31
ASA	2.6	0,5	2.2	0.8	0.33
Therapy strategy	1.4	0.5	1.5	0.7	0.45
Construct length [N/segments]	2.5	0.7	2.2	0.5	0.09
Follow-up time [months]	53.5	27.0	45.0	23.0	0.23

*Std* Short Standard deviation, Trauma mechanism: 1: not memorable, 2: low energy trauma; 3: moderate energy trauma; 4: high energy trauma; Fracture location: 1: L1; 2: L2; 3: Th 3; 4: Th4; 5: Th5; 6; Th6; 7: Th7; 8: Th8; 9: Th9; 10: Th10; 11: Th11; 12: Th12; 13: L3; Fracture classification: 1: type OF 1; 2: type OF 2; 3: type OF 3; 4 type OF 4; 5: type OF 5; ASA: Therapy strategy: 1: Hybrid stabilization (posterior stabilization + kyphoplasty of the fractured vertebral body; 2: posterior stabilization only; 3: combined anterior and posterior stabilization; American Society of Anesthesiologists; N/segments: number of segments

## Discussion

The first finding of this study is that subsequent vertebral body fractures were associated with significant worse outcome regarding the ODI score, the pain level and the general well-being (PSC – SF36). The second finding is that subsequent vertebral body fractures were significantly more frequent in patients with not remembered trauma. Five out of six patients non-memorable trauma suffered from a subsequent fracture in the further course.

Interestingly, the huge majority of patients with subsequent vertebral body fractures were female with only one male patient being affected. This ratio was significant different compared to the ratio of the included patient population. Additionally, the patient collective with subsequent vertebral body fractures had a significantly higher loss of reduction. Thereby, the mean difference of loss of reduction is only 2°. This is caused by the inclusion of patients without loss of reduction of 10° or more. This seems to underline the fact that fracture reduction is important and the reduction loss should ideally be kept as small as possible. Thereby, the authors believe, that the amount of reduction loss plays an important role for the occurrence of subsequent fractures even though the reduction loss is small to moderate. No signs of re-fracture or implant loosening were seen at the fractured vertebral body and the region of stabilization. In this context, Okamoto et al. [8] compared the effect of a kyphotic deformity of 10° versus 20° of the level of T12 and found an increased stress at the adjacent vertebral body by a factor of 2 and 4 as well as a bimodal peak of the stress at the midthoracic spine. This was the reason to define the acceptable bisegmental loss of reduction of less than 10° as long as sufficient reduction was achieved. Thereby, it is important to respect the physiological alignment of the geriatric spines.

The physiological alignment of the spine changes during the course of aging. This associated with an increased thoracic kyphosis, a decreased lumbal lordosis and sacral slope, as well as an increased C7 sagittal vertical axis [13]. Thereby, the origin of sagittal deterioration has been discussed controversially [14, 15]. Generally, the differentiation between physiologic and pathologic alignment can be difficult in the elderly population. However, a deteriorated alignment is associated with reduced quality of life [16]. This needs to be avoided as long as possible.

Generally, gentle reduction by ideal patient positioning and gentle distraction force are recommendable. However, powerful reduction maneuvers should be avoided potentially causing implant failure in the further course. In fact, those patients without subsequent vertebral fractures had very promising results with a mean ODI score of 18% despite of a median age of 71 years at the time of surgery and a mean follow-up of close to four years. Similarly, Gu et a [17] reported of improved regional alignment in patients with unstable osteoporotic thoracolumbar vertebral body fractures treated by hybrid stabilization compared to cement augmentation only. Thereby, the reduction potential was similar between both techniques. However, the loss of reduction was significantly higher in patients treated only with kyphoplasty or vertebroplasty leading to noticable higher numbers of subsequent vertebral body fractures.

Another risk factor for adjacent fractures might be the cement augmented screws which might be responsible of higher grades of degeneration at the adjacent intervertebral discs increasing the stress on the adjacent vertebral bodies [18, 19]. However, this argument cannot explain the high number of subsequent fractures of vertebral bodies not adjacent to the instrumentation.

The very high rate of subsequent vertebral body fractures in patients without remembered trauma and the high rate of subsequent fractures after low-energy trauma lead to the question if insufficiency fractures are comparable to osteoporotic vertebral body fractures caused by moderate or high energy traumas. The comparability of these fractures seems to be limited. This could explain the wide range of adjacent vertebral fractures in the further course both after nonoperative and operative treatment depending on the included patient population [20]. Clinical results improve tremendously by including only patients with moderate and high energy trauma. This patient group had a mean ODI score of 18.8 compared to 53.0 in patients, who don’t remember any trauma. Interestingly, patients with low-energy trauma had comparable ODI-scores to those patients with moderate and high energy trauma despite the far higher rate of subsequent vertebral body fractures. Generally, it is necessary to discuss the indication for surgery and the applied technique. Whereas the huge majority of patients with low-, moderate and high energy trauma did well and seemed to benefit from surgery using hybrid stabilization or by a combined anterior–posterior approach, patients without remembered trauma might have not gained relevant improvement by hybrid stabilization or surgery at all. It would be a very interesting study question to compare the outcome of patients with unstable insufficiency fractures at the thoracolumbar spine after the treatment with hybrid stabilization with either cement augmentation only or with non-operative treatment strategies.

Altogether, this study offers several limitations. First of all, the retrospective study design has to be discussed critically. This includes the potential bias caused by five different implants by four manufacturers and different treatment strategies that have been used. Additionally, patients that have been included might differ tremendously in respect of the individual bone quality. Some of the patients suffered from insufficiency fractures whereas others had a history of moderate to high energy trauma. Next, the clinical scores that have been used can be biased by other pathologies particularly as no comparison measurement was available based on the retrospective study design. Furthermore, only patients treated with posterior stabilization with or without cement augmentation of the fractured vertebral body or a combined anterior approach were included. Thereby, Spiegl et al. [21] reported of similar clinical und radiological results between hybrid stabilization and combined anterior–posterior surgery in elderly patients. Generally, the objective for including patients with posterior instrumentation with or without anterior fusion were the unexpected high rates of patients with subsequent vertebral fractures and unsatisfying outcomes in patients with incomplete vertebral body fractures in elderly patients treated with posterior stabilization despite low rates of reduction losses [6]. Thereby, three different strategies including hybrid stabilization, long-segmental posterior stabilization and combined anterior and posterior stabilization were included. This leads to a selection bias. However, the main inclusion criterium was posterior stabilization in contrast to conservative treatment and cement augmentation only. Additionally, no bone density scan was done as a routine practice during that time period. Thus, the severity of the individual osteoporosis could not be estimated, and no anti-osteoporotic therapy was started in the majority of the patients during the hospital stay, even though a clear recommendation was given for it in the discharge report. However, in accordance with the literature, this leads to high rates of insufficient osteoporotic diagnosis and treatment which affects the outcome of this study. Generally, analysis of the hounsfield unites of the posttraumatic CTs would have been very interesting [22, 23]. However, in several patients the CTs were performed at the primary hospital prior transferring the patient for surgical treatment. Those CTs were stored for a restricted time period and were not available at the time of data analysis. Additionally, the rate of patients that received a correct anti-osteoporotic therapy is unknown. Some of the subsequent vertebral body fractured could have been avoided in those patients that did not receive correct anti-osteoporotic treatment. Based on this we changed our strategy and perform a DEXA-scan during the hospital stay and start with the specific antiosteoporotic therapy before dismissal. However, based on the fact that the majority of subsequent fractures are occurring during the first year [24] and the delayed effect of fracture reduction using bisphosphonates mainly after the first 6 to 12 months of treatment [25], this might have not such a great influence on the number of subsequent fractures. Last but not least, the number of patients without remembered trauma is only small. Notwithstanding, five out of six of those suffered a subsequent vertebral fracture what is statistically significant.

Altogether, the high number of patients with unstable fractures of the thoracolumbar spine in geriatric patients and the long median follow-up period are the strengths of this study.

## Conclusion

Patients with subsequent vertebral fractures had a significantly inferior midterm outcome. Thereby, the trauma mechanism correlated significantly with both the rate of subsequent vertebral body fractures and the outcome. Particularly, patients with non-memorable trauma had high rates of subsequent fractures. Another risk factor is female gender.

## Data Availability

The datasets generated analyzed during the study are available from the corresponding author on reasonable request.
